# Introducing integrated laboratory classes in a PBL curriculum: impact on student’s learning and satisfaction

**DOI:** 10.1186/1472-6920-13-71

**Published:** 2013-05-24

**Authors:** Samy A Azer, Rana Hasanato, Sami Al-Nassar, Ali Somily, Muslim M AlSaadi

**Affiliations:** 1 Medical Education and Curriculum Development & Research Unit, College of Medicine, King Saud University, P O Box 2925, Riyadh 11461, Saudi Arabia; 2 Department of Pathology, King Saud University, P O Box 2925, Riyadh 11461, Saudi Arabia; 3 Department of Medical Education, King Saud University, P O Box 2925, Riyadh 11461, Saudi Arabia; 4 Department of Paediatrics, College of Medicine, King Saud University, P O Box 2925, Riyadh 11461, Saudi Arabia

**Keywords:** Medical education, Integrated laboratory classes, Students’ learning, Impact on learning, Laboratory procedures

## Abstract

**Background:**

With the introduction of integrated problem-based learning (PBL) program in the medical curriculum, there is a need to create laboratory classes that suit students’ learning needs and the changes introduced to the curriculum. This paper outlines the development and implementation of four integrated laboratory classes (ILCs) at King Saud University College of Medicine. It also examines whether core concepts addressed in these classes were learned and retained and how the students perceived the ILCs.

**Methods:**

ILCs are based on enhancing enquiry-based learning, and encouraging students to work on tasks in small groups (apply and integrate knowledge from biochemistry, pathology and microbiology) and conduct a laboratory procedure (practical part). In two of these ILCs, a pretest comprising 15 multiple-choice questions were administrated at the start of the class and an identical posttest was administrated at the end of these classes. Performance of the students in the Objective Structured Practical Examination (OSPE) at the end of the blocks was also evaluated. Students’ perceptions were evaluated using a questionnaire completed at the end of each class.

**Results:**

A total of 247, 252, 238, and 244 students participated in practical classes covering cerebrospinal fluid infection, small intestine, liver function tests and adrenal gland function, respectively. Students got higher scores in posttests compared to pre-test scores in two classes (12.68 ± 2.03 vs 6.58 ± 3.39 and 13.02 ± 2.03 vs 7.43 ± 2.68, respectively). Paired t-test showed that the difference was significant (P < 0.001) in both tests. The mean scores of students in stations dealing with ILCs at the end of the block examinations were not significantly different from the mean scores for other stations not related to ILCs. The questionnaire indicated that most students expressed positive attitude towards working on tasks and applying knowledge learnt. Students also felt that conducting laboratory procedures and interpreting laboratory findings were valuable to their learning.

**Conclusions:**

Given the increase in the posttest scores (short-term retention) and the satisfactory performance of students at the end of block examinations (long-term retention) together with the students’ satisfaction, the study suggests that the core concepts addressed in these classes were learned and retained.

## Background

If medical education aims at deep understanding of concepts, development of competencies, and application of knowledge, new learning modalities should be introduced. Over the last three decades several changes have been introduced to medical education worldwide. These changes were initiated in response to several declarations including the General Medical Council “Tomorrow’s Doctors” [1], the Australian Medical Council [2-4], the Association of American Medical Colleges (AAMC), and the National Commission for Academic Accreditation & Assessment [5]. The aims of these recommendations are: making more emphasis on concepts and principles and moving from an overloaded medical curriculum, introducing integration across basic and clinical sciences, enhancing application of knowledge, and changing examinations from focusing on memorization of factual knowledge to testing competencies, cognitive skills, application of knowledge, and deep understanding [6].

King Saud University (KSU) College of Medicine, a leading medical college in Saudi Arabia, has introduced a hybrid problem-based learning (PBL) into the preclinical years. These changes commenced in the academic year 2009/2010. The rationale for the changes introduced in the medical curriculum are (i) a traditional curriculum may enables students to understand basic sciences such as anatomy, physiology, pathology, pharmacology etc., but does not prepare students for the clinical years and learning in a clinical environment, (ii) the explosion in medical/health information and the rapid progress of research and discoveries necessitates that medical curricula focus on learning approaches that enhance student’s self-directed learning, and life-long learning, (iii) medical workforces are looking for doctors who are competent, and have acquired knowledge, skills, and professional attitude required for clinical practice; an established traditional curriculum usually focus on acquisition of medical knowledge and memorisation of facts and therefore does not prepare graduates to fulfil the needs of the medical workforce, and (iv) medical graduates usually travel seeking postgraduate education and clinical practice in other countries, therefore good universities are usually interested in reshaping their curricula to meet international standards and contribute to the profession. Based on these changes, the college adopted changes that are based on best practices and evidence from medical education research.

KSU College of Medicine accepts students after completing a one-year course comprising subjects such as biology, chemistry, physics, medical biostatistics, and English for medical profession. Only students with a grade point average (GPA) higher than 3 are accepted [7].The new medical curriculum strongly emphasizes the importance of small group learning, problem-based learning, self-directed learning, enquiry-based learning, use of e-learning, and hands-on training in clinical skills lab, as well as practical classes. While some medical schools choose to cluster PBL cases around themes such as “oxygen delivery”, “disturbed consciousness”, “life cycle” etc. with less strict boundaries to body systems [8], The curriculum development unit decided to organize the PBL cases around parallel body systems [9,10] with the opportunity for students to revisit such themes and build on them from cases allocated to different body systems [11]. In the medical program, the preclinical years are represented in the first two years where disciplines are integrated in a block/module system. The blocks in these two years are: Foundation block, Musculoskeletal block, Respiratory block, Cardiovascular block, Renal block, Nervous System block, Gastrointestinal and Haematology block, Endocrine block and Reproduction block. The remaining three years revolve around various clinical clerkships, and preparation of students to join the medical workforce. To achieve these goals, a new department for medical education was established in 2009, with five specialized units: curriculum development unit, assessment and evaluation unit, clinical skills unit, research and dissemination unit, and Information technology unit. These units work together in harmony with the other 19 departments in the college of medicine to design and implement the new medical curriculum.

Laboratory classes offer an opportunity for enforcing learning and complementing other teaching modalities such as problem-based learning (PBL), lecture, self-directed learning, and e-learning. While PBL and lectures usually focus on the big picture, laboratory classes facilitate learning about details and hands-on experiences. Ideally laboratory classes should parallel the changes introduced to the curriculum-enable students to apply knowledge learnt, discover relationships, conduct laboratory procedures, and end with a meaningful learning [12-14].

### Rationales for ILCs

The changes introduced to certain practical classes are a direct response to students and staff difficulties with the understanding the role of preclinical laboratory classes in an integrated curriculum and how these classes should address student’s learning needs in an engaging manner. The ILCs aimed at replacing certain practical classes in which students investigated a concept using the microscope, laboratory animals, or laboratory procedures [12]. In most schools using traditional laboratory classes, students are given a laboratory guidebook in which the procedures are precisely described, and students merely follow the guidebook without thinking deeply [13,14]. With the changes introduced to laboratory classes worldwide, to reduce animal experimentation, these classes were replaced by class demonstrations and then by a video showing an experiment conducted in a class. These changes limited the value of laboratory classes and encouraged passive learning [12,15]. In the meantime, knowledge and skills learnt from each laboratory class such as biochemistry, pathology, and microbiology were discipline-based and did not enable students to examine integration of such skills or correlate learning outcomes from such classes to clinical applications. There is no doubt that these classes were useful in assisting students to develop their experimental techniques, observational skills and laboratory skills. However, traditional practical classes were not successful in assisting students to understand application of knowledge learnt and realize the value of basic sciences in clinical investigations conducted for clinical diagnosis of diseases. Also non-integrated classes did not bridge the gap between what students learn from problem-based learning tutorials, lectures, and small group tutorials with desired laboratory skills. Therefore, in the development process of these ILCs, an important starting point was the realization that preclinical classes need to be student-centered, address the outcomes of the curriculum and match with the changes introduced in the curriculum. However, not all practical classes can be integrated due to the nature of their contents and educational objectives. Such practical classes were left as discipline based classes.

### ILCs target students’ learning needs

In the early stages of designing the ILCs it was decided to identify the learning needs of the students. Our analysis of students’ performance in year 1 examinations (midblock and the end of the block examinations) showed that students have difficulties in integrating knowledge and applying knowledge learnt to clinical situations. Skills in areas such as interpreting laboratory results and using the findings to refine their hypotheses and correlate biochemical changes with likely pathological and microbiological changes were deficient. Students also experienced difficulties in understanding concepts such as pathogenesis of diseases and signaling sequences of biochemical changes that could end in pathological changes. Such deficiencies and learning difficulties highlight the needs for further training in applying knowledge learnt and correlating microbiology, biochemistry and pathology to clinical situations. The analysis also helped us to select task-based and student-centered approaches in developing these laboratory classes with an emphasis on cognitive outcomes, procedural skills and application of knowledge. This analysis forms the basis on which we introduced the ILCs to fill the gap in the curriculum and help students to overcome these learning difficulties.

With the introduction of the ILCs it was necessary to assess the impact of these classes on students’ learning. To assess the impact of a new teaching/learning modality on student’s learning, it may be necessary to assess short- and long-term retention as well as feedback provided by the learners on their experience [16-18]. For example, in what way has the new teaching/learning modality helped them, has these classes added to their learning experience, and learner’s suggestions for further improvement of such modality. Therefore, this paper aims at outlining the development and implementation of four integrated laboratory classes (ILCs) at King Saud University College of Medicine examining whether core concepts addressed in these classes were learned and retained (on short and long-term bases) and how the students perceived the learning new modality.

## Methods

### Organisation of the laboratory activities

Although the biochemistry, pathology, and microbiology units at the College of Medicine have been clustered under the department of pathology for a number of years, this is the first time for the three units to work together with the department of medical education to create such initiative. The laboratory classes used in the curriculum were mainly focused on a particular discipline, and did not link with the theme of the week or provide students with meaningful learning. Also there were no specific tasks needed from students to work on. In these classes, students observed a demonstration/experiment conducted on a laboratory animal, examine some slides under the microscope, or conduct an experiment [19].

The concept of integrated laboratory classes was new to academics. The department of medical education worked on facilitating the design and the educational framework of these laboratory classes. The aims were to create new laboratory classes that suited the changes introduced to the medical curriculum and based on educational objectives. However, not all practical classes can be integrated due to the nature of content and educational objectives. Such classes were left as disciple-based classes. The design is focused on student-centered learning [20], fit with the theme of the week, reflect integration, and provide students with meaningful learning. The creation of such laboratory classes was achieved through three key steps: Identification, preparation, and implementation (Table 1). First, the identification of a particular laboratory class that can be integrated- this is the responsibility of the block committee members. The team decides on suggesting a particular integrated class if the class fits with the theme of the week, enables students to better understand an important concept in the curriculum, and allows them to examine the application and different aspects of a concept. For example, an integrated laboratory class on liver function tests in the gastrointestinal block that can be placed in the week covering the hepatobiliary system. Second, preparation-this is the responsibility of 3–4 academics representing the units/departments involved in each laboratory together with a member from the curriculum development unit. Members meet 4–5 times to prepare the learning outcomes of the laboratory, the tasks needed from students to work on during the class, the laboratory procedures to be conducted by students, and the discussion part at the end of the class. Third, implementation-the success of this part is dependent on the preparation part and the effectiveness of the team involved in following the schedule placed for the laboratory class and keeping these classes student-centered. All facilitators and lab assistants involved are asked to attend a final meeting and are briefed about the laboratory class (Table 1).

**Table 1:** Steps for organizing an integrated laboratory classes

**Step 1: Identification**	**Step 2: Preparation**	**Step 3: Implementation**
The committee members of each block identify topics/areas that can be taught in integrated practical classes.	For each integrated practical class, the working group has to meet approximately 4–5 times to prepare for a practical class. Usually they start meeting about 4–5 weeks prior to the date of the class.	Each practical class is 2-hour long and is conducted three times to ensure that the maximum number of students attending is about 90 students. Students are then allocated into two equal groups, Groups A and B, of 45 students each.
The organizing working group for each practical class comprises an academic from the biochemistry, microbiology, pathology and medical education disciplines.	The preparation and organization covers the following areas:	Group A students started in the Pathology Laboratory at Level 1 and worked on case scenarios for one hour. The case scenarios aimed at providing students with skills to integrate knowledge from biochemistry, microbiology, and pathology related to topic covered.
	• Defining the learning outcomes of the practical class.	Group B students started in the Multipurpose Laboratory at level 2 and worked on a laboratory procedure related to the practical class for one hour (e.g., measurement of serum bilirubin). Students were then asked to switch to the other Lab to complete the second task for another one hour.
	• Designing the educational components and tasks to be completed by students.	At the end of each component, students discussed the tasks with their tutors and received feedback. At the end of the practical class, students were asked to complete a questionnaire.
	• Organising the sequence of the practical class activities, and time allocated for each activity, discussion, the procedure component, and feedback on tasks.	In some practical classes, a pre- and post-test comprising 15 single-best MCQs were endorsed. Students were asked to answer all questions at the beginning of the practical class and then the same questions, but in a different order, were answered by students at the end of the class.
	• Organising the purchase of kits, calibrating equipment, and preparing the experimental protocol.	
	• Piloting, for some practical classes, to ensure successful implementation. Ensuring that all facilitators are briefed about the practical class and understand their role.	
	• Preparing students’ hands-out, instructions, tasks, case scenarios, biochemistry lab results, pathology and microbiology image to be included in the tasks.	
	• Allocating students into groups and logistics for the design of the practical class.	

#### Objectives and pedagogy behind ILCs

The ILCs are designed with the following objectives in mind:

– Providing students with the opportunity to apply knowledge learnt by integrating knowledge across disciplines.

– Facilitating learning through small group discussion and enquiry-based learning.

– Encouraging students to interpret biochemical test results and pathological findings and link their findings to case scenarios and use evidence in refining their hypotheses.

– Enhancing learning through laboratory procedure skills and introducing students to laboratory techniques.

– Enabling students to explore meaningful learning by examining the biochemical, pathological, and microbiological aspects of common diseases from the laboratory aspects.

Therefore, the objectives and design of ILCs are different from teacher-centered approaches that aim at the transmission of factual knowledge to the learners and turning learning into memorization of pieces of information.

### Task-based learning and procedure learning

**Figure 1:**
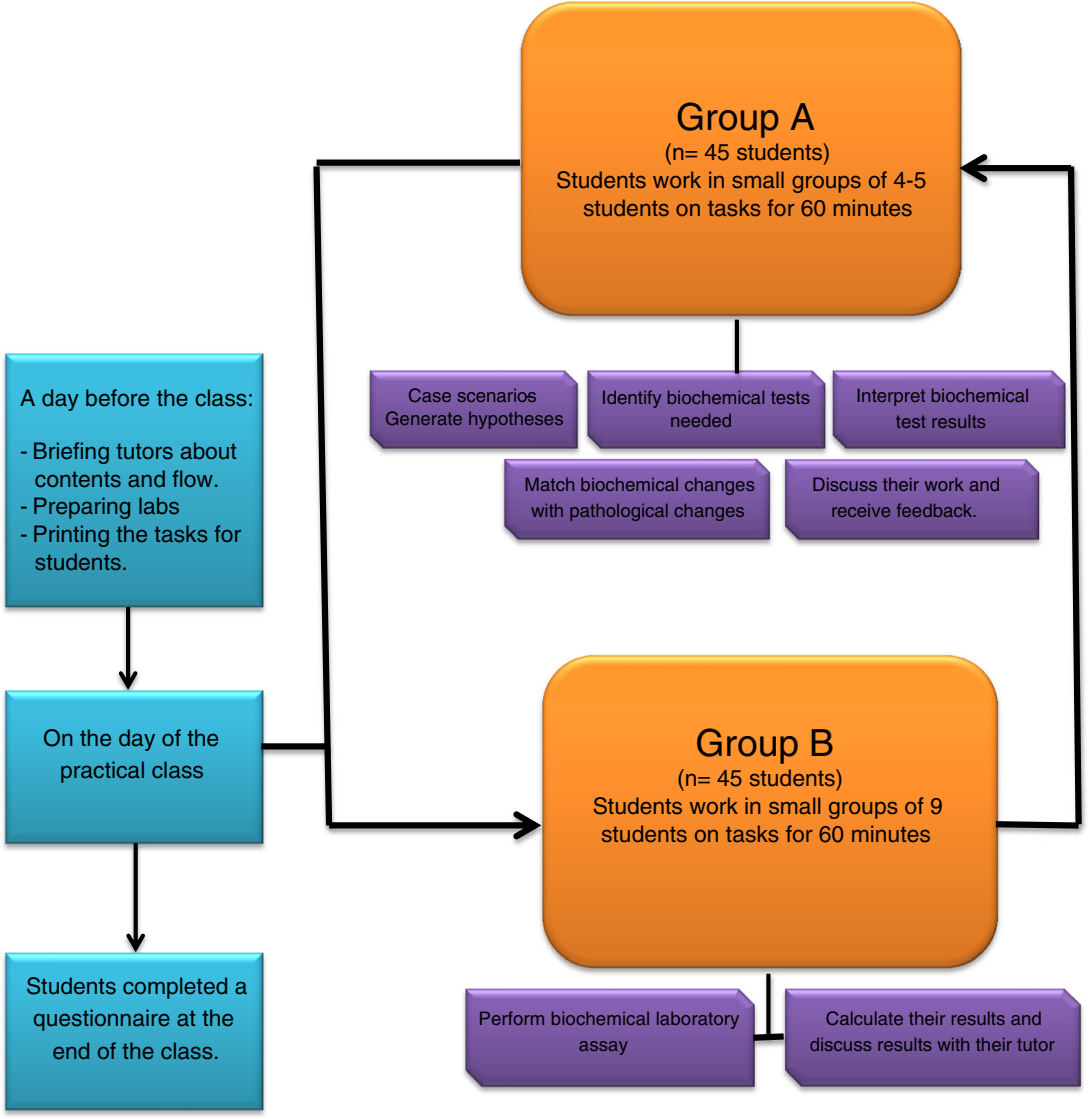
Implementation of an integrated practical class.

To achieve the objectives, it is important to design these classes in a way that enables students to work in small groups of 3–4 students to complete specific tasks [20]. The tasks included in ILCs include: brief case scenarios reflecting the objectives of the laboratory class and stimulating students to interpret biochemical laboratory test results provided with the case, identify further investigations needed, interpret microbiological tests, describe the pathological changes in a slides/images related to the case, match the pathological changes with the findings obtained from biochemical test results, as well as perform a laboratory procedure that match with the objectives of the laboratory classes. Therefore the cognitive skills embedded in the laboratory tasks are consistent with the pedagogy of PBL and reinforce the curriculum design. The learning objectives of each ILC and the tasks given to the students are summarized in Figure 1 and Table 2.

**Table 2:** Themes, learning objectives and tasks included in integrated laboratory classes

**Block**	**Week**	**Theme**	**Integrated disciplines**	**Learning objectives**	**Tasks/ Lab procedure**
Nervous System Block	6	Cerebrospinal fluid infection (CSF)	Biochemistry	• State the biochemical and microbiological tests used in CSF examination.	• Interpret the biochemical results of a CSF examination for three different cases.
			Microbiology	• Practice reading laboratory reports and learn how to interpret findings and link their interpretation to case scenarios.	• Identify the possible infection responsible and justify their views.
			Medical Education	• Demonstrate the ability to look into evidence to justify their views.	• Discuss further laboratory investigations needed.
				• Discuss the scope of different biochemical, pathological, and microbiological tests used and significance of the results.	• Practice measurement of glucose level in a CSF sample and compare their readings.
				• Learn key laboratory skills related to CSF examination.	
Gastrointestinal and Haematology Block	2	Small intestine and the pancreas	Biochemistry	• State the biochemical tests used in assessment of patients with malabsorption (steatorrhoea).	• Interpret the biochemical results of reports for four different cases with pancreatic problems and malabsorption.
			Pathology	• Practice reading laboratory reports and learn how to interpret findings and link their interpretation to case scenarios.	• Discuss further investigations needed.
			Medical Education	• Demonstrate the ability to look into evidence to justify their views.	• Identify the possible cause responsible and justify their views.
				Discuss the scope of different biochemical, and pathological tests used and significance of the results.	• Practice measurement of serum amylase and compare their readings.
				• Learn key laboratory skills related to measurement of serum amylase.	
Gastrointestinal and Haematology Block	4	Liver function tests	Biochemistry	• State the biochemical and microbiological tests used in assessing liver functions.	• Interpret the biochemical results of liver function tests for four different liver cases.
			Pathology	• Practice reading laboratory reports and learn how to interpret findings and link their interpretation to case scenarios.	• Identify the possible infection/aetiology responsible and justify their views.
			Microbiology	• Demonstrate the ability to look into evidence to justify their views.	• Discuss further laboratory investigations needed.
			Medical Education	• Discuss the scope of different biochemical, pathological, and microbiological tests used in assessing liver functions and significance of the results.	• Practice measurement of serum bilirubin level and compare their readings.
				• Learn key laboratory skills related to liver function tests.	
Endocrine Block	4	Adrenal function	Biochemistry	• State the biochemical tests used in assessing adrenal function.	• Interpret the biochemical results of adrenal function tests for four different cases.
			Pathology	• Practice reading laboratory reports and learn how to interpret findings and link their interpretation to case scenarios.	• Identify the possible aetiology responsible and justify their views.
			Medical Education	• Demonstrate the ability to look into evidence to justify their views.	• Discuss further laboratory investigations needed.
				• Discuss the scope of different biochemical, and pathological changes and significance of the results.	Practice measurement of serum cortisol level and compare their readings.
				Learn key laboratory skills related to adrenal function tests.	

### Study design

Table 2 summarises four ILCs introduced in the new medical curriculum. Each of these classes are 2 hours long. To ensure that students’ number for each laboratory class is in the range of 80–90 students, it is decided to conduct each laboratory class three times. Students are then divided as per the protocol of the integrated laboratory classes into two equal groups, Groups A and B, with each group comprising 45 students are allocated into a laboratory class facility with tutors. In each class, students in small groups of 3–4 work together on tasks. Tutors from biochemistry, microbiology and pathology are allocated to these classes and this has necessitated preparing all tutors and briefing them about their roles during the preparation stage.

### Short-term retention

To examine basic information and student’s skills in microbiology, pathology, and biochemistry dealing with an ILC, all students underwent a pretest as they start the class. A test comprises 15 multiple-choice questions (MCQs) with a single best answer out of 5 options. The aim of such tests is to explore student’s knowledge and skills related to the practical class prior and after the completion of the class immediately (learning and short term retention). Neither student’s name nor student’s ID were identified at the test. On the pretest paper a random code number was given and students were asked to remember their number and write the same number on their posttest paper. Students were given 15 minutes to complete the test. Test papers were collected by the tutors before the start of the laboratory class. At the end of the preclinical class, students took the same test and their scores were compared.

### Long-term retention

To assess the impact of the four ILCs on learning and long-term retention and whether students learned how to apply knowledge in an integrated way, students’ scores in the end of the block Objective Structured Practical Examination (OSPE) for each block were evaluated and compared with students’ scores in other stations for non-integrated laboratory classes.

### Students’ evaluation of ILCs

This study was designed to include all preclinical-years students. At the end of each laboratory class, students were invited to complete closed ended items on a five-point Likert scale questionnaire [21] where 1 indicated “strongly agree” and 5 indicated “strongly disagree”. In the questionnaire, students were asked to make a valued judgment in relation to these main areas: i) demographic data (gender, and age), ii) the value of integration across disciplines in IPCs to their learning, iii) perceptions regarding the use of cases, and laboratory test results in IPCs, iv) perceptions about working with others in small groups in IPCs, and v) the value of discussion and receiving feedback at the end of the laboratory class. The last section of the questionnaire comprised open-ended questions with the aim to evaluate the overall students’ views about the whole laboratory class. The following open-ended statements were mentioned, ‘I believe that the best aspects of the laboratory class are…..”, “I believe the laboratory class can be improved by considering the following suggestions”, and “My other comments are….”. A cover letter addressing the aims of the questionnaire and confidentiality issues was distributed along with the questionnaire. The questionnaire was based on our prior experience in this area [22,23] and was piloted in 2010 before its use in 2011 and 2012. The study was approved by the Department of Medical Education and the College of Medicine. Data were collected over a period of two years.

### Statistical analysis

All analyses were performed using SPSS for Windows 14.0 -SPSS 2010 [24]. The chi-square test was used to analyse differences in nominal data, while the Mann–Whitney U test was employed for ordinal data [15,25,26]. Cronbach’s alpha was employed to assess the internal consistency of the subscales of the instrument [27]. Qualitative comments were typed out and repeated themes in the data were identified. A paired t-test and ANOVA were used to analyze pretest and posttest results. A *p* value of < 0.05 was considered significant.

## Results

### Short-term retention

In two integrated laboratory classes covering liver function tests (practical 3) and adrenal function (practical 4) students got higher scores in their posttest of knowledge as compared to their pretest 12.68 ±2.03 vs 6.58 ± 3.39 and 13.02 ±2.03 vs 7.43 ± 2.68, respectively. The results were significantly different (P < 0.001) in both classes (Table 3). No tests were conducted for the first and second practical classes as we were more concerned about the design of our new model and students’ satisfaction.

**Table 3:** Pre- and post-test scores in two integrated laboratory classes

**Integrated laboratory class**	n*	**Pre-test (mean ± SD)**	**Post-test (mean ± SD)**	***t***	***P *value**
Practical 3: Liver Function Tests	202	6.58 ± 3.39	12.68 ±2.03	+34.105	<0.001
Practical 4: Adrenal Function	206	7.43 ± 2.68	13.02 ±2.03	+41.03	<0.001

n* = number of students completed and retuned both the pre- and post-test answers. Paired t-test is used in the analysis. The level of significance was set at *P* < 0.05.

### Long-term retention

As shown in Table 4, students did not perform at a satisfactory level in the first OSPE following the introduced of ILCs in 2010/2011. The mean scores in a station covering cerebrospinal fluid infection were significantly lower compared to their performance in other stations related to non-integrated classes (1.32 ± 0.58 vs 4.08 ± 1.97; p < 0.009, respectively). However, students’ performance in the OSPE in the following blocks in stations related to ILCs was significantly improved in years 2010/2011 and in 2011/2012. As shown in Table 4, the means of students’ scores in stations related to ILCs were not significantly different from those obtained in other stations covering concepts discussed in non-integrated classes. This indicates the ability of students to comprehend complex integrated tasks and retain such skills on long-term-bases.

**Table 4:** Summarising students’ performance in the end of block Objective Structured Practical Examination (OSPE) in stations dealing with concepts covered in ILCs vs scores from stations covering concepts discussed in non-integrated classes

**Block**	**Concept covered**	**Academic year 2010/2011**			**Academic year 2011/2012**		
		**Station number**	**Scores in stations covering ILCs mean ± SD**	**Scores in stations not related to ILCs mean ± SD**	**Station number**	**Scores in stations covering ILCs mean ± SD**	**Scores in stations not related to ILCs mean ± SD**
Nervous system block	Microbiology, Biochemistry & Pathology	12	1.32 ± 0.58	4.08 ± 1.97*	14 and 15	7.59 ± 2.02	8.06 ± 1.12**
						8.26 ± 2.67	
Gastrointestinal & Haematology block	Microbiology, Biochemistry & Pathology	3, 7, 9 10, 11 and 12	9.08 ± 1.55	8.22 ± 1.40***	5, 6, and 9	8.69 ± 2.00	8.21 ± 1.06^#^
			9.55 ± 2.02			8.01 ± 1.45	
			8.82 ± 1.83			8.94 ± 1.92	
			9.24 ± 1.76				
			9.54 ± 1.24				
			6.99 ± 3.61				
Endocrine block	Biochemistry & Pathology	12 and 13	9.74 ± 0.89	9.87 ± 0.76^##^	3 and 15	9.82 ± 0.92	9.84 ± 0.15^$^
			7.47 ± 1.56			9.22 ± 1.33	

Means (SD) are shown over two academic years in three blocks.

*P value < 0.009; **P value < 0.420; ***P value <0.342; ^#^P value <0.299; ^##^P value <0.301; ^$^p value < 0.392.

### Students’ evaluation of ILCs

The results of the students’ feedback about four laboratory classes are summarized in Table 5. Perceptions were evaluated on the basis of gender and no significant differences were found. The response rates were 93% for the first laboratory class, 95% for the second, 89% for the third class, and 92% for the fourth class. The percentages of participants parallel the gender distribution of students in the college in these years. The Cronbach alpha values varied between 0.70 and 0.86 which are within the preferable range [28].

**Table 5:** Students’ views about integrated laboratory classes

**Question**	Responses*	**Practical 1: CSF infection**	**Practical 2: Small intestine and pancreas**	**Practical 3: Liver function tests**	**Practical 4: Adrenal gland function**
		**Number (%)**	**Number (%)**	**Number (%)**	**Number (%)**
Q1: Overall I enjoyed working on cases to apply knowledge learnt.	Strongly Agree	151 (61.1)	118 (46.8)	117 (49.2)	108 (44.3)
	Agree	84 (34.0)	107 (42.5)	102 (42.9)	108 (44.3)
	Unable to decide	7 (2.8)	12 (4.8)	15 (6.3)	23 (9.4)
	Disagree	3 (1.2)	1 (0.4)	0 (0.0)	3 (1.2)
	Strongly disagree	2 (0.8)	3 (1.2)	4 (1.7)	2 (0.8)
	Total number	247 (100)	252 (100)	238 (100)	244 (100)
Q2: The feedback from the tutor at the end was useful to my learning.	Strongly Agree	133 (53.8)	106 (42.1)	96 (40.3)	92 (37.7)
	Agree	90 (36.4)	116 (46.0)	104 (43.7)	108 (44.3)
	Unable to decide	19 (7.7)	16 (6.3)	29 (12.2)	34 (13.9)
	Disagree	3 (1.2)	1 (0.4)	5 (2.1)	8 (3.3)
	Strongly disagree	2 (0.8)	2 (0.8)	4 (1.7)	2 (0.8)
	Total number	247 (100)	252 (100)	238 (100)	244 (100)
Q3: The integration of subjects such as biochemistry, pathology, and microbiology in this practical class helped me to better understand issues related in the practical class.	Strongly Agree	139 (56.3)	110 (43.7)	83 (34.9)	92 (37.7)
	Agree	87 (35.2)	100 (39.7)	99 (41.6)	98 (40.2)
	Unable to decide	16 (6.5)	20 (7.9)	41 (17.2)	44 (18.0)
	Disagree	4 (1.6)	9 (3.6)	11 (4.6)	7 (2.9)
	Strongly disagree	1 (0.4)	2 (0.8)	4 (1.7)	3 (1.2)
	Total number	247 (100)	252 (100)	238 (100)	244 (100)
Q4: The laboratory procedure conducted was useful to my learning**	Strongly Agree	102 (41.3)	113 (44.8)	79 (33.2)	73 (29.9)
	Agree	92 (37.2)	86 (34.1)	115 (48.3)	114 (46.7)
	Unable to decide	40 (16.2)	30 (11.9)	27 (11.3)	41 (16.8)
	Disagree	10 (4.0)	11 (4.4)	14 (5.9)	11 (4.5)
	Strongly disagree	3 (1.2)	1 (0.4)	3 (1.3)	5 (2.0)
	Total number	247 (100)	252 (100)	238 (100)	244 (100)
Q5: I enjoyed working with and learning from other students in this practical class.	Strongly Agree	115 (46.6)	103 (40.9)	90 (37.8)	94 (38.5)
	Agree	104 (42.1)	117 (46.4)	103 (43.3)	97 (39.8)
	Unable to decide	25 (10.1)	18 (7.1)	32 (13.4)	38 (15.6)
	Disagree	2 (0.8)	1 (0.4)	8 (3.4)	12 (4.9)
	Strongly disagree	1 (0.4)	2 (0.8)	5 (2.1)	3 (1.2)
	Total number	247 (100)	525 (100)	238 (100)	244 (100)
Q6: Practical classes should have cases to work on, followed by tutor discussion of tasks provided and hands-on training related to the practical class.	Strongly Agree	134 (54.3)	112 (44.4)	97 (40.8)	103 (42.2)
	Agree	89 (36.0)	102 (40.5)	99 (41.6)	89 (36.5)
	Unable to decide	15 (6.1)	20 (7.9)	33 (13.9)	39 (16.0)
	Disagree	7 (2.8)	3 (1.2)	4 (1.7)	10 (4.1)
	Strongly disagree	2 (0.8)	4 (1.6)	5 (2.1)	3 (1.2)
	Total number	247 (100)	252 (100)	238 (100)	244 (100)
Q7: I learnt better through working on tasks and working with others	Strongly Agree	102 (41.3)	69 (27.4)	75 (31.5)	77 (31.6)
	Agree	103 (41.7)	116 (46.0)	110 (46.2)	106 (43.4)
	Unable to decide	27 (10.9)	38 (15.1)	31 (13.0	32 (13.1)
	Disagree	10 (4.0)	10 (4.0)	13 (5.5)	22 (9.0)
	Strongly disagree	5 (2.0)	8 (3.2)	9 (3.8)	7 (2.9)
	Total number	247 (100.0)	252 (100)	238 (100)	244 (100)
Q8: My learning is enhanced when I use several resources (e.g., multimedia, PBL cases, practical classes, and textbooks).	Strongly Agree	93 (37.7)	71 (28.2 )	65 (27.3)	78 (32.0)
	Agree	108 (43.7)	107 (42.5)	107 (45.0)	99 (40.6)
	Unable to decide	32 (13.0)	41 (16.3)	41 (17.2)	36 (14.8)
	Disagree	6 (2.4)	17 (6.7)	16 (6.7)	18 (7.4)
	Strongly disagree	8 (3.2)	5 (2.0)	9 (3.8)	13 (5.3)
	Total number	247 (100.0)	252 (100)	238 (100)	244 (100)
Q9: I learn better by hands-on working on activities in practical classes	Strongly Agree	139 (52.6)	104 (41.3)	82 (33.2)	95 (38.9)
	Agree	87 (39.3)	115 (45.6)	111 (46.6)	105 (43.0)
	Unable to decide	13 (5.3)	18 (7.1)	31 (13.0)	27 (11.1)
	Disagree	4 (1.6)	2 (0.8)	12 (5.0)	15 (6.1)
	Strongly disagree	3 (1.2)	2 (0.8)	2 (0.8)	2 (0.8)
	Total number	247 (100)	252 (100)	238 (100)	244 (100)

* A 5-point Likert scale, where 1 indicated “strongly agree”, 3 indicated “unable to decide”, and 5 “strongly disagree”.

** The laboratory procedure was for Practical 1: measurement of glucose concentration in a cerebrospinal fluid sample, for Practical 2: measurement of serum amylase activity, for Practical 3: measurement of serum bilirubin concentration, Practical 4: measurement of serum cortisol.

### Qualitative comments

Not all participants completed the commentary parts in the questionnaire. We include here the comments obtained from the first three classes. Only 59%, 79%, and 61% of those returning the questionnaire answered the open-ended questions in the questionnaire collected at the end of each of the first three practical classes, respectively. In most cases not all open-ended questions were completed. These comments were grouped and summarized as follows (i) the case scenarios and the tasks were useful to my learning and application of knowledge learnt from lectures and PBL (83%), (ii) the laboratory procedures enabled us to experience laboratory skills (89%), (iii) the discussion and feedback at the end of the practical class were useful (74%), (iv) Compared to traditional classes, the structure and design of the laboratory class were engaging (79%), (v) the classes allowed me to work with other students (66%), and (vi) researching and discussing the tasks by students were useful (58%).

## Discussion

This study aimed to evaluate four ILCs introduced for the first time in our college and to assess the impact of such teaching/learning modality on students’ learning; short and long-term retention. Overall, students’ feedback on the ILCs was very high and most encouraging. The study also showed that students’ learning in the new design fostered self-directed learning, integration of knowledge and learning. This has been demonstrated in short-term retention (pretest and posttest results) and long-term retention (performance in the OSPE at the end of the block). The study also showed that the use of task-based learning, small group discussion, case scenarios, biochemistry and microbiology laboratory results, together with pathology sections and laboratory procedures enabled students to integrate knowledge and develop meaningful learning.

The topics selected for integrated laboratory classes were chosen on the bases of clinical significance, lack of clarity in the old curriculum, ensuring that the laboratory class enforces integration across basic sciences, and that laboratory classes fit with the theme of the week. However, not all practical classes fulfilled the purpose of integration and were not changed. Student’s active participation in laboratory work enhanced the learning processes [20] and facilitated storage of new knowledge into memories. Enforcement of learning by doing encourages the learner to build confidence during episodic retrieval of remembered information [29]. On neurobiological basis, there is evidence that active engagement of the learner is associated with changes in neural circuitry that are associated with learning. Learning in small groups is associated with the opportunity for learners’ contribution, personal accountability, and collaboration. It also turns learning into an enjoyable experience. This is particularly reinforced when the learners are asked to work in small groups to complete a task within a specified time and report back to their findings. Receiving feedback on tasks completed enables students to learn from their mistakes, build their confidence in what they know, and add to their learning experiences. Such an approach encourages learners to interact, ask questions, raise hypotheses, search for answers, and examine their abilities to justify their views. This is different from a teacher-centered approach that aims at transmission of factual knowledge to the learners and turning learning into memorization of pieces of information [13,30].

In addition to learning by doing and active participation [31], ILCs enabled practice and visualisation of procedures and integration of knowledge from biochemistry, microbiology, and pathology. Such integration is important for understanding the biochemical and pathological basis of mechanisms by which diseases occur and how different disease processes are associated with different pathological changes. The results of this study showed that students’ performance in OSPE stations related to ILCs were not significantly different from their performance in simple non-integrated stations indicating their long-term retention of complex learning tasks. Although students struggled with such higher ordered learning tasks in their first OSPE following the introduction of ILCs, such challenge disappeared as demonstrated from their performance in integrated station in several blocks in years 2010/2011 and 2011/2012 (Table 4).

The use of case scenarios allowed learners to think, visualize changes and link knowledge from several disciplines to answer challenges/tasks and justify their views. It is obvious from the students’ responses to the questionnaire that they enjoyed learning in the new format [32]. The use of case scenarios, and exposing students to biochemical, microbiology, and pathological changes in relation to these scenarios allowed students to put different pieces of knowledge together, justify their views, and apply knowledge learnt to real life situations. It also enabled them to practice the measurement of serum bilirubin, serum amylase, and cerebrospinal glucose concentration, and understand the scientific basis behind the procedure, and calculate their concentrations in biological samples. One of the limitations of this study is that the results about the learning effects in ILCs is not compared to a control group for whom the same amount of time was spent, but in another format. Such design may be difficult to implement for several logistic factors. However, we have examined the long-term retention of concepts learned in ILCs through their performance in the OSPE stations and compared the performance of students in such stations with their performance in other stations covering concepts discussed in non-integrated practical classes. Such comparison has added important aspect to the impact of ILCs on students’ learning.

## Conclusions

In conclusion, given the increase in the posttest scores (short-term retention) and the satisfactory performance of students at the end of block examinations (long-term retention), together with the satisfactory feedback from students, the study suggests that the core concepts addressed in these classes were learned and retained.

## Competing interests

The authors report no conflicts of interest. The authors alone are responsible for the content and writing of the paper.

## Authors’ contribution

SAA, initiated the idea of integrated practical classes, led the team in designing the four practical classes, and writing the material needed, contributed to the statistical analysis, and the writing of the paper and tables, RH, contributed to the writing of the material needed for the practical classes, successful implementation of these practical classes, collection of data, and contributed to writing the paper, SAN, contributed to the writing of the paper, AS, contributed to the writing of the material needed for the practical classes, successful implementation of these practical classes, collection of data, statistical analysis and contributed to the writing of the paper, MMA, contributed to the writing of the paper. All authors read and approved the final manuscript.

## Authors’ information

Samy A. Azer, MD, PhD (USyd), MEd (UNSW), FACG (USA), MPH (UNSW) is Professor of Medical Education and the Head of Curriculum Development and Research Unit, College of Medicine, King Saud University. He was Professor of Medical Education and Chair of Medical Education Research and Development Unit, Faculty of Medicine, Universiti Teknologi MARA, Malaysia. Formerly he was Senior Lecturer in Medical Education at the Faculty of Medicine, Dentistry and Health Sciences, the University of Melbourne and the University of Sydney, Australia.

Rana Hasanato, MD, is Assistant Professor in Biochemistry, Department of Pathology at College of Medicine, King Saud University and Head of the Biochemistry Unit at King Khalid Hospitals, Riyadh, Saudi Arabia.

Sami Al-Nassar, MD, FRCSC is Assistant Professor of Surgery, Consultant Thoracic Surgeon, and Chairman of the Department of Medical Education, College of Medicine, King Saud University, Riyadh, Saudi Arabia.

Ali Somily, MD, is Assistant Professor in Microbiology, Department of Pathology at College of Medicine, King Saud University and Head of the Microbiology Unit at King Khalid Hospitals, Riyadh, Saudi Arabia.

Muslim M AlSaadi, MD, ABP, FCCP, is Consultant and Professor of Paediatrics and Vice Dean for Academic Affairs, College of Medicine, King Saud University.

## Pre-publication history

The pre-publication history for this paper can be accessed here:

http://www.biomedcentral.com/1472-6920/13/71/prepub

## Support

This work was funded by College of Medicine Research Center, Deanship of Scientific Research, King Saud University, Riyadh, Saudi Arabia.

## Conference presentations

Parts of this study were presented at the Australian & New Zealand Association for Health Professional Educators (ANZAHPE), Rotorua, New Zealand from 26 to 29 June 2012 and as short communication at the Association for Medical Education in Europe (AMEE), Lyon, France from 25 to 29 August 2012.
